# Diversity and Distribution of Fungi in the Marine Sediments of Zhanjiang Bay, China

**DOI:** 10.3390/jof10120867

**Published:** 2024-12-13

**Authors:** Menghan Gao, Bihong Liu, Jianming Li, Yunyan Deng, Yulei Zhang, Ning Zhang, Feng Li, Changling Li, Xianghu Huang, Zhangxi Hu

**Affiliations:** 1 Guangdong Provincial Key Laboratory of Aquatic Animal Disease Control and Healthy Culture, College of Fisheries, Guangdong Ocean University, Zhanjiang 524088, China; gaomenghan0914@163.com (M.G.); 2112101021@stu.gdou.edu.cn (B.L.); 13434643014@stu.gdou.edu.cn (J.L.); zhangyl@gdou.edu.cn (Y.Z.); zhangn@gdou.edu.cn (N.Z.); lifeng2318@gdou.edu.cn (F.L.); licl@gdou.edu.cn (C.L.); huangxh@gdou.edu.cn (X.H.); 2 Guangdong Laboratory of Marine Ecology Environment Monitoring and Warning, Guangdong Ocean University, Zhanjiang 524088, China; 3 CAS Key Laboratory of Marine Ecology and Environmental Sciences, Institute of Oceanology, Chinese Academy of Sciences, Qingdao 266071, China; yunyandeng@qdio.ac.cn; 4 Laboratory for Marine Ecology and Environmental Science, Qingdao Marine Science and Technology Center, Qingdao 266071, China

**Keywords:** Zhanjiang Bay, fungal community, diversity, FUNGuild, molecular ecological network, environmental factors

## Abstract

Fungi are one of the major components of the eukaryotic microbial community in marine ecosystems, playing a significant role in organic matter cycling and food web dynamics. However, the diversity and roles of fungi in marine sediments remain poorly documented. To elucidate the diversity and spatial distribution of fungal communities in the marine sediments of an estuary–coast continuum across three distinct salinity regions in Zhanjiang Bay, China, the variations in fungal diversity, abundance, community structure, and distribution in the sediments were investigated through the application of high-throughput amplicon sequencing using the internal transcribed spacer (ITS) primers. Additionally, the FUNGuild database was employed to assess the potential functional traits of fungi. A total of 1242 ASV sequences, affiliated to 144 genera and five phyla, were identified. Ascomycota (68.97%) and Basidiomycota (6.41%) were the dominant fungal groups, together accounting for 75.38% of the total relative abundance of the fungal community. Significant differences were observed in the α-diversity indices (Shannon index and richness) and β-diversity of fungal communities across the three distinct salinity regions. The fungal molecular network exhibited primarily positive species interactions, with notable structural differences across salinity gradients. The low-salinity group had a large network with high modularity; the medium-salinity group a small, simple network with high centralization, and the high-salinity group a compact, moderately complex network. Symbiotrophs, saprotrophs, and pathotrophs, being the three trophic types with the highest proportions, were estimated based on ITS. A redundancy analysis (RDA) indicated that salinity was the primary factor influencing the distribution of Ascomycota communities, while the distributions of Basidiomycota, Chytridiomycota, Mucoromycota, and Rozellomycota were more strongly affected by environmental factors such as chlorophyll *a*, chemical oxygen demand (COD), pH, and temperature. Our work provides new scientific data on the diversity, composition, and distribution of fungal communities in Zhanjiang Bay, which helps to understand the biodiversity of fungi in the estuary–coast ecosystems.

## 1. Introduction

Microbial communities are the primary drivers of nutrient and energy cycling in aquatic sediments, with fungi representing a crucial component of these communities [1]. Fungi are widely distributed across various marine ecosystems, including open waters, deep-sea anoxic environments, algal beds, etc. [2]. They play multifaceted roles within the food web, functioning as producers and decomposers, and existing in diverse forms such as saprophytes, parasites, and symbionts [3]. Through the production of potent extracellular enzymes, fungi are capable of degrading a wide range of organic matter, thereby contributing to nutrient provision, enhancing the physicochemical properties of sediments, and mitigating the stress induced by pollutants such as heavy metals [4]. Moreover, fungi are instrumental in nutrient transformation, cycling, and energy flow within sedimentary environments [5,6]. Despite their ecological significance, research on fungal communities in aquatic environments remains relatively limited, especially when compared to the extensive studies on bacterial and archaeal community structures [7]. This gap highlights the need for further investigation into the roles and dynamics of fungal communities in these ecosystems.

In recent decades, human activities have increasingly impacted coastal waters, leading to significant environmental changes [8]. The continuous influx of nutrients, such as nitrogen and phosphorus, into the ocean has caused a notable increase in nutrient concentrations in coastal water, resulting in widespread eutrophication across global coastal regions [9,10]. Marine fungal communities, being highly sensitive to ecological shifts, are particularly susceptible to these alterations [11,12,13]. Numerous studies have demonstrated that factors such as temperature, salinity, pH, and nutrient levels can significantly influence the structure and diversity of these communities [14,15]. Rising temperatures generally stimulate the proliferation of the fungi typically found in warmer waters, while also potentially intensifying the eutrophication process, which in turn can lead to shifts in fungal community composition. Furthermore, temperature variations may influence the degradation capacity of fungi, thereby affecting water quality and nutrient cycling dynamics. For example, Tisthammer et al. investigated the fungal community structure in the surface seawater of Helgoland Roads and found that temperature and chlorophyll *a* were critical environmental factors affecting fungal diversity in this area [16]. Fluctuations in chlorophyll *a* concentrations are intricately linked to the growth and decay of phytoplankton, which provide a primary source of organic matter for fungi in aquatic ecosystems. As such, variations in chlorophyll *a* levels can exert a significant influence on both the community structure and functional dynamics of fungal populations. Salinity is another critical environmental factor that governs the growth and distribution of marine fungi, with different fungal species exhibiting varying degrees of tolerance to salinity levels. Consequently, changes in salinity within marine ecosystems can drive shifts in the relative abundance and dominance of specific fungal species. The existing research indicates that the distribution of marine fungi in the Guangdong coastline is closely associated with nutrient levels and salinity [17]. Rojas-Jimenez et al. analyzed the diversity and composition of fungal communities in the Baltic Sea by 18S rRNA gene sequencing and found significant differences in fungal diversity and distribution across various salinity gradients [18]. Similarly, Burgard et al. found that low-salinity environments substantially affected the fungal communities in estuaries and bays [19]. Additionally, Gilbert et al. examined the seasonal distribution patterns of fungal communities in the English Channel, and identified temperature, nitrate, and silicate concentrations as the key factors shaping fungal community structure [20]. Further research has indicated that eutrophication can profoundly impact fungal community structure and diversity. For instance, Sen et al. analyzed fungal communities in Shenzhen Bay, Daya Bay, and the South China Sea, and discovered that increasing eutrophication led to a gradual decline in the relative abundance of Basidiomycota, with pH and temperature also playing significant roles in influencing fungal diversity [21]. Zhao et al. observed that as nutrient concentrations increased, the abundance of two Ascomycota taxa, each with an initial average abundance of less than 0.01%, increased significantly. They further concluded that nutrient enrichment intensified interspecies interactions by alleviating resource competition and providing a broader range of resources for certain taxa [22]. Collectively, these studies highlight the fact that fluctuations in temperature and the progression of eutrophication not only have a profound impact on the composition of fungal communities but also influence the overall functionality of marine ecosystems. This occurs through the alteration of resource availability and modifications to interspecies competition dynamics. Given these findings, investigating the structures and influencing factors of fungal communities in marine environments is of critical importance for predicting the responses of marine ecosystems to ongoing environmental changes.

Zhanjiang Bay, located on the northeastern side of the Leizhou Peninsula in the western Guangdong province, China, is adjacent to the South China Sea. Covering a water area of 1419 km^2^ with an average depth of approximately 13 m, the bay is encircled by numerous industrial zones, agricultural regions, aquaculture farms, ports, and densely populated residential areas [23]. There are 11 municipal wastewater discharge outlets along the coastline of Zhanjiang Bay, through which over 2 million tons of industrial, agricultural, and domestic wastewater are released annually. This discharge introduces substantial amounts of nutrients and organic matter, which, in turn, influence hydrological parameters such as pH, nutrient concentrations, temperature, and dissolved oxygen levels in the seawater, either directly or indirectly [24]. These alterations in environmental conditions can significantly impact the biological communities within the marine ecosystem and contribute to the establishment of diverse microbial resources in the region. However, most previous research has primarily concentrated on issues related to heavy metal pollution [25], sources of organic contaminants [26], water quality, and environmental monitoring [27], with relatively limited attention given to the fungal community structure and the processes governing its development in this marine environment. Marine fungi play a vital role in maintaining the health and ecological balance of estuarine ecosystems. For instance, they are involved in the degradation of anthropogenic pollutants, such as polyethylene [28], petroleum hydrocarbons [29], and the bioaccumulation of heavy metal ions [30], thereby contributing to water purification and ecological restoration processes. Moreover, fungi have been shown to be more effective than bacteria in some cases for the bioremediation of heavy metals [31]. In addition, fungi in estuarine environments can form symbiotic relationships with plants, such as seaweed [32] and mangroves [33], enhancing the host’s stress tolerance and facilitating the uptake of essential nutrients like nitrogen and phosphorus. Given these ecological functions, it is essential to gain a comprehensive understanding of the fungal community structure and its temporal and spatial distribution patterns in Zhanjiang Bay. Therefore, this study aims to analyze the fungal species diversity and community structure in the sediments of Zhanjiang Bay through high-throughput sequencing using the internal transcribed spacer (ITS: a non-coding region of DNA located between ribosomal RNA (rRNA) genes in the genome) primers and bioinformatics approaches. To the best of our knowledge, this is the first detailed report on the fungal diversity in the sediments of Zhanjiang Bay, providing new scientific data for the study of fungal diversity in Zhanjiang Bay, as well as a new scientific basis for follow-up studies on the correlation between estuarine–coastal fungal communities and environmental factors. It also lays the theoretical foundation for investigating the ecological roles of fungi in this habitat.

## 2. Materials and Methods

### 2.1. Sampling Sites and Sediment Sample Collection

**Figure 1:**
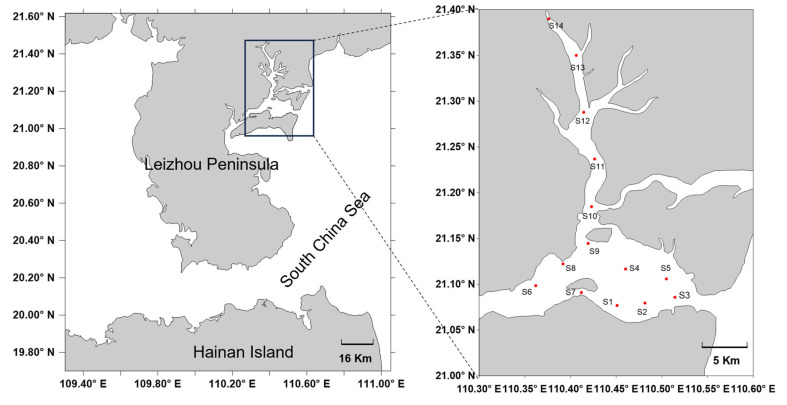
Location of the sampling sites in the Zhanjiang Bay, China.

Zhanjiang Bay is located in Zhanjiang city, Guangdong province, China, and it is a semi-enclosed bay receiving freshwater from the upstream and directly connecting to the South China Sea in the southeast part (Figure 1). Fourteen surface sediment samples (0–3 cm) were collected from 14 sampling sites (high salinity: S1, S2, S3, S4, and S5; medium salinity: S6, S7, S8, and S9; and low salinity: S10, S11, S12, S13, and S14) using a grab sampler in April 2023. Approximately 10 g of the top 3 cm sediment samples was sub-sampled by a manual core from each site and stored in the dark at 4 °C until further analyses.

### 2.2. Environmental Factor Analyses

In situ, a multi-parameter water quality analyzer (LH-T600, Zhejiang Lohand Environment Technology Co., Ltd., Hangzhou, China) was employed to measure various parameters of surface water (0–1.0 m) at each station, including water temperature, salinity, pH, dissolved oxygen (DO), and chlorophyll *a* content. Subsequently, 10 L of surface water was collected using a water sampler. The water samples were filtered through a 0.45 µm cellulose acetate membrane, and the filtrates were used for nutrient analyses. The concentrations of NO_3_-N, NO_2_-N, NH_4_-N, PO_4_-P, and SiO_3_-Si were determined using a Smart 200 automatic nutrient analyzer (ASM, Milano, Italy). Chemical oxygen demand (COD) was measured by the alkaline potassium permanganate method [34]. The concentration of dissolved inorganic nitrogen (DIN) was calculated as the sum of NO_3_-N, NO_2_-N, and NH_4_-N.

### 2.3. DNA Extraction, PCR Amplification, ITS Sequencing, Data Processing, and Bioinformatic Analyses

The total genomic DNA in sediment was extracted using the Power Soil DNA Isolation kit (MP Biomedicals, Santa Ana, CA, USA) according to the manufacturer’s protocol. Nuclear-free water processed through DNA extraction was used as the sample blank. The concentration and gross mass of DNA were quantified using a NanoDrop^TM^ 1000 spectrophotometer (Thermo Fisher Scientific, Waltham, MA, USA). The ITS2 region was amplified using the primers fITS7 (5′-GTGARTCATCGAATCTTTG-3′) [35] and ITS4 (5′-TCCTCCGCTTATTGATATGC-3′) [36] under the following thermal cycling conditions: initial denaturation at 98 °C for 30 s, followed by 32 cycles of denaturation at 98 °C for 10 s, annealing at 54 °C for 30 s, and extension at 72 °C for 45 s, with a final extension at 72 °C for 10 min. PCR amplification was performed in a 25 μL reaction mixture containing 25 ng of template DNA, 12.5 μL of PCR Premix, 2.5 μL of each primer, and PCR-grade water to achieve the final volume. The 5′ ends of the primers were tagged with sample-specific barcodes and universal sequencing primers. The integrity of the PCR products was confirmed through electrophoresis on a 2% agarose gel. Throughout the DNA extraction process, nuclease-free water was used as a negative control, replacing the sample solution to minimize the risk of false-positive PCR results. After amplification, the PCR products were purified using AMPure XT beads (Beckman Coulter Genomics, Danvers, MA, USA) and quantified with a Qubit fluorometer (Invitrogen, Waltham, MA, USA). The qualified PCR products were evaluated using an Agilent 2100 Bioanalyzer (Agilent, Santa Clara, CA, USA) and Illumina library quantitative kits (Kapa Biosciences, Woburn, MA, USA). Sequencing was performed on the Illumina NovaSeq 6000 (PE250) (LC-Bio Technology Company, Hangzhou, China).

The sequencing primers were removed from de-multiplexed raw sequences using cutadapt (v1.9). Then, paired-end reads were merged using FLASH (v1.2.8). The low-quality reads (quality scores < 20), short reads (<100 bp), and reads containing more than 5% “N” records were trimmed by using the sliding-window algorithm method in fqtrim (v 0.94). Quality filtering was performed to obtain high-quality clean tags according to fqtrim (v 0.94). Chimeric sequences were filtered using the Vsearch software (v2.3.4). DADA2 was applied for denoising and generating amplicon sequence variants (ASVs). The taxonomy of the ASVs was assigned using the consensus blast method of the “feature-classifier” plugin in QIIME 2 (https://qiime2.org/, accessed on 8 May 2023) [37] against the UNITE dynamic database [38,39]. The relative abundance of each ASV was estimated based on its read counts normalized to the total number of good-quality reads.

### 2.4. Data Analyses

The α-diversity indices of fungal communities in marine sediment, including the Shannon index and richness index, were calculated using the “vegan” package in the R software (v.4.1.2). Non-metric multidimensional scaling (NMDS) based on Bray–Curtis and Jaccard distance was employed to assess the β-diversity of fungal communities. Phylogenetic trees, accompanied by species-level bubble diagrams, were constructed using the Autools platform (https://www.cloudtutu.com, accessed on 15 October 2023). A redundancy analysis (RDA) was conducted using the “vegan” package in R (v.4.1.2) to evaluate the correlation between the fungal community structure and environmental factors. The co-occurrence networks of fungi in the sediment at the phylum level (IRI > 0.7, *p* < 0.01) were visualized using the SparCC method with the “igraph” package and Gephi (v.0.9.7) interactive software. Functional predictions for fungal communities were carried out using the FUNGuild database (http://www.stbates.org/guilds/app.php, accessed on 20 October 2023).

### 2.5. Accession Numbers for Nucleotide Sequences

All the sequence data were deposited in the Sequence Read Archive of the NCBI under accession number PRJNA1173321.

## 3. Results

### 3.1. Distribution Characteristics of Environmental Factors in Zhanjiang Bay

The water depth ranged from 2 to 37 m (Table 1). The water temperature (24.08–25.13 °C) and pH (7.61–7.72) exhibited minimal fluctuations across the study area (Table 1). The salinity ranged from 18.48 to 32.07, generally displaying a spatially decreasing trend from the southern to the northern regions of the bay and from the bay mouth toward the inner bay (Table 1). The dissolved inorganic nitrogen (DIN) concentrations fluctuated from 0.209 to 0.770 mg/L, with an average of 0.313 mg/L (Table 1). The phosphate levels ranged from 0.002 to 0.117 mg/L, with an average concentration of 0.044 mg/L (Table 1). The concentrations of silicate varied between 1.385 and 5.428 mg/L, with an average of 2.007 mg/L (Table 1). The chemical oxygen demand (COD) concentrations ranged from 0.880 to 2.960 mg/L, with an average of 1.320 mg/L (Table 1). Spatially, all four factors—DIN, phosphate, silicate, and COD—exhibited a trend of gradually increasing from the southern to the northern regions of the bay and from the bay mouth toward the inner bay.

### 3.2. Composition and Distribution of Fungi in the Sediments of Zhanjiang Bay

**Figure 2:**
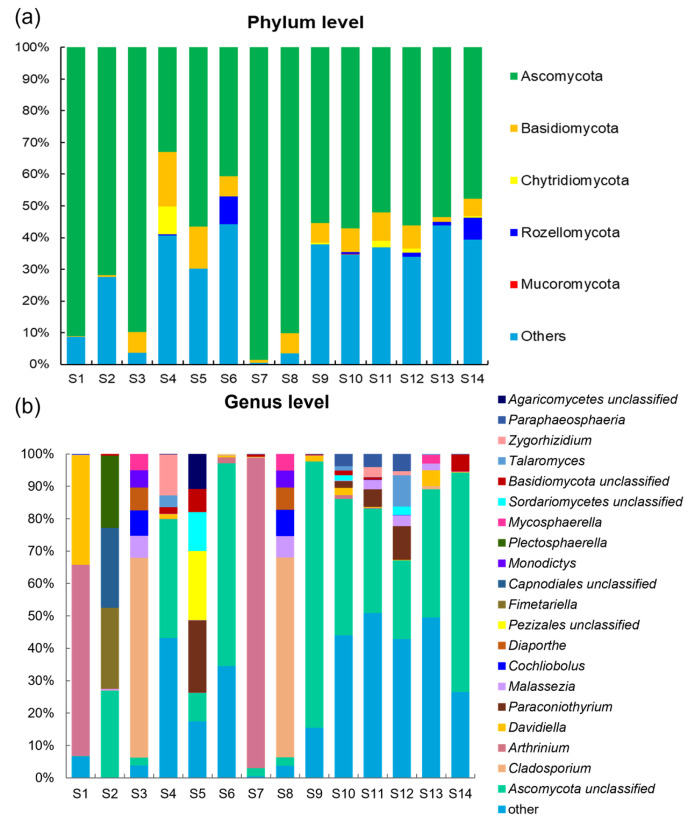
Species composition and relative abundance of fungal communities in sediments of Zhanjiang Bay, China ((**a**): phylum level; (**b**): genus level; other: fungi unclassified).

ITS sequencing was conducted on sediment fungal communities from the 14 stations in Zhanjiang Bay, China. A total of 1,044,244 raw rDNA sequence reads from the 14 samples corresponded to 0.51 Gb of raw data, with the sequences per sample ranging from 51,264 to 87,121 (Supplementary Table S1). The denoising of the raw reads using the DADA2 plugin within the QIIME 2 tool resulted in 887,194 effective sequences, which were then binned into 1242 amplicon sequencing variants (ASVs). Based on the analyses of species abundance and annotation, a total of 144 genera and five phyla were identified (Figure 2). In the taxonomic hierarchy, species that could not be definitively identified or those with relatively low abundance were classified as “other”. As a result, 23.01% and 16.97% of the fungi at the phylum and genera levels, respectively, were categorized under “other” (Figure 2).

The whole fungal microbiome consisted of Ascomycota (68.97%), Basidiomycota (6.41%), Chytridiomycota (0.85%), and Rozellomycota (0.75%), with rare occurrence of Mucoromycota (0.01%) (Figure 2a). The ASVs within these five phyla were further classified into 23 classes, 55 orders, 94 families, and 144 genera (Supplementary Tables S3 and S4). At the phylum level, Ascomycota was the most dominant fungal phylum, representing 33.03–98.53% (Figure 2a). The relative abundance of Ascomycota was notably higher at stations S1, S2, S3, S7, and S8 than that in the other sites. Ascomycetes was the second most dominant fungi in the sample. Additionally, Ascomycota and Basidiomycota were present at all the sites; Rozellomycota was only found at stations S4, S6, S10, S12, S13, and S14; while Mucoromycota was exclusively detected at station S10. Conversely, the relative abundance of the “other” category at stations S1, S2, S3, S7, and S8 was significantly lower than that at the other stations (Figure 2a).

The species composition across different sites exhibited significant variation at the genus level, particularly in response to differing salinity conditions (Figure 2b). Under high-salinity conditions, the dominant taxa were primarily from the Ascomycota phylum, including Ascomycota unclassified, *Cladosporium*, *Arthrinium*, *Davidiella*, and *Fimetariella*. Notably, Ascomycota unclassified dominated at the sites S2 and S4, with a relative abundance of 36.65% at site S4. *Cladosporium* was the most prevalent genus at site S3, with a striking relative abundance of 61.63%, while at site S1, *Davidiella* and *Arthrinium* contributed significantly to the community composition (Figure 2b). In the medium-salinity group, the dominant genera were Ascomycota unclassified, *Arthrinium*, and *Cladosporium* (Figure 2b). Ascomycota unclassified showed predominance at sites S6 and S9, with relative abundances of 62.65% and 82.24%, respectively. *Arthrinium* was more abundant at the site S7. Similarly, *Cladosporium* exhibited a notable presence at site S8, where its relative abundance reached 61.75%. In the low-salinity group, Ascomycota unclassified, *Paraconiothyrium*, and *Malassezia* dominated, while other genera were present at lower abundances. Ascomycota unclassified was particularly dominant at sites S9 and S14, where its relative abundance exceeded 67%. The overall species diversity in the low-salinity group was relatively low, while the relative abundance of the “other” taxa in the low-salinity group was significantly higher compared to the other salinity groups. Across all the salinity conditions, unclassified Ascomycota remained the predominant genus. While *Cladosporium* and *Arthrinium* were dominant in the high- and medium-salinity environments, their prevalence declined in low-salinity conditions. Notably, Pezizales unclassified was found exclusively in the high-salinity group, with a considerable relative abundance of 21.37% at site S5. *Fimetariella* was present solely at site S2, albeit at low abundance, while *Diaporthe* occurred exclusively in the medium- and high-salinity groups, specifically at sites S3 and S8.

Additionally, a significant proportion of fungal sequences could not be annotated at the species level due to the limited sequence information available in the current databases. This limitation arises primarily because some fungi were difficult to collect and culture, making it challenging to obtain the necessary identification data.

### 3.3. Diversity of Fungal Communities in the Sediments of Zhanjiang Bay, China

**Figure 3:**
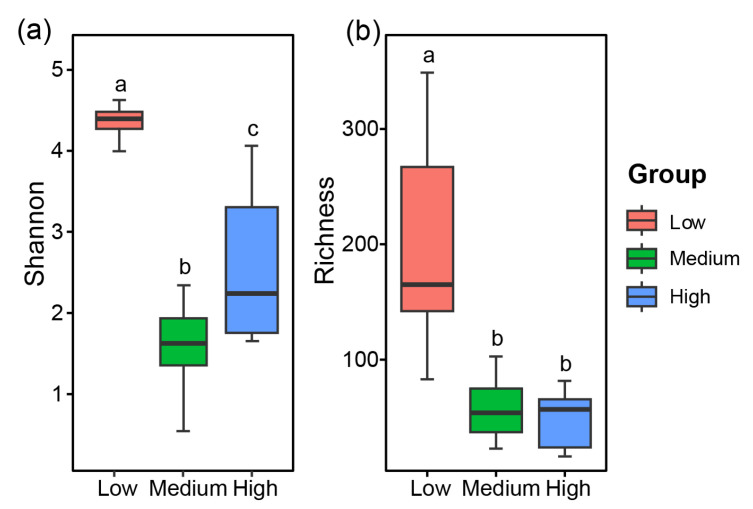
The α-diversity analysis of the fungal communities in the sediments of Zhanjiang Bay, China (Shannon (**a**) and Richness (**b**); different lowercase letters represent significant differences (*p* < 0.05), and the same lowercase letters no significant differences (*p* ≥ 0.05). Low: low salinity; Medium: medium salinity; High: high salinity).

Both the Shannon index and Richness index were significantly higher in the low-salinity group than those in the other two groups (*p* < 0.05; Figure 3a). The Shannon index exhibited the pattern being low-salinity group > high-salinity group > medium-salinity group, with statistically significant differences observed among the three groups (*p* < 0.05; Figure 3a). Regarding the Richness index, a significant difference was evident between the low-salinity group and the other two groups (*p* < 0.05), whereas the difference between the high- and medium-salinity groups was not statistically different (*p* > 0.05; Figure 3b).

**Figure 4:**
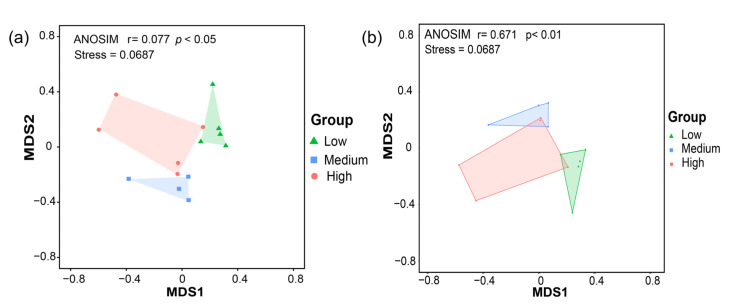
The β-diversity of the fungal communities in the sediments of Zhanjiang Bay, China (NMDS analysis using (**a**) Bray–Curtis and (**b**) Jaccard distances. Low: low salinity; Medium: medium salinity; High: high salinity).

The similarity of the fungal communities in the sediments from different stations in Zhanjiang Bay was evaluated using non-metric multidimensional scaling (NMDS) based on Bray–Curtis and Jaccard distances (Figure 4). A stress value below 0.2 indicates that the NMDS plot provides a reliable representation of the data. An R value greater than 0 suggests effective grouping, while a p value less than 0.05 denotes a statistically significant difference. The NMDS analysis revealed that the composition of sediment fungal communities differed significantly across the three salinity zones (*p* < 0.05; Figure 4).

### 3.4. Molecular Ecological Network Analysis of Fungal Communities

**Figure 5:**
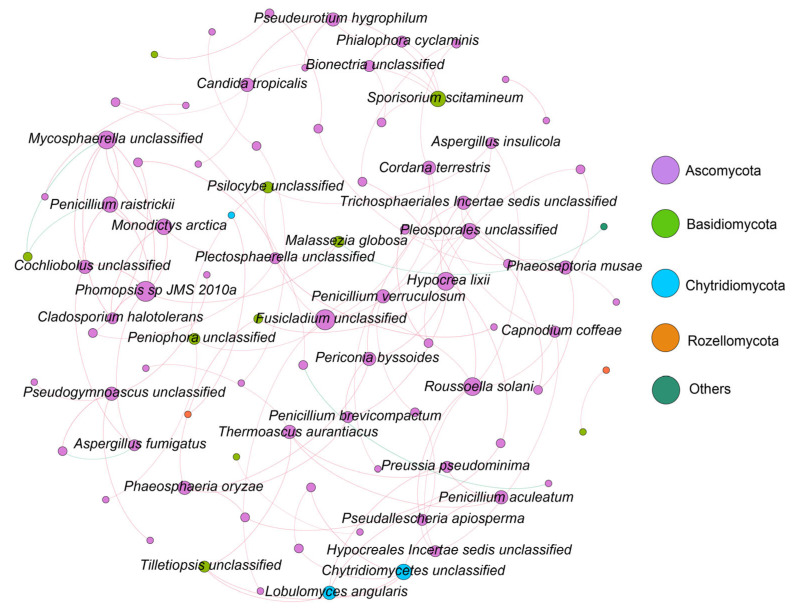
Molecular ecological network of fungal communities in the sediments of Zhanjiang Bay, China (Each node represents a species, with node size proportional to connectivity. Red edges indicate positive correlations, while green edges indicate negative correlations).

Molecular ecological networks at the phylum level were constructed for the fungal communities in Zhanjiang Bay (Figure 5). The results indicated that the primary fungal phyla involved in the network connections were Ascomycota, Basidiomycota, Chytridiomycota, and Rozellomycota, while the other fungal phyla and unannotated species contributed relatively little to the network construction (Figure 5). Ascomycota emerged as the dominant phylum across all four molecular ecological networks, which were characterized by predominantly positive correlations among the interacting species (Figure 5 and Supplementary Figures S7–S9). The network topological properties revealed significant differences among the salinity groups. The network in the low-salinity group comprised 57 nodes and 56 edges, while the medium-salinity group 16 nodes and 17 edges, and the high-salinity group 23 nodes and 21 edges (Supplementary Table S5). Additionally, the average degree in the low-salinity group (1.965) was lower than that of both the medium- (2.125) and the high-salinity groups (2.598). The network density in the low-salinity group was also lower than that in the medium- and high-salinity groups. In contrast, the molecular modularity of the fungal community in the low-salinity group (0.845) was higher than that in the medium- (0.428) and high- (0.834) salinity groups (Supplementary Table S5). In summary, the networks of the low-, medium-, and high-salinity groups exhibited distinct characteristics (Supplementary Figures S7–S9). The network in the low-salinity group was larger and more dispersed, yet it demonstrated a higher molecular modularity. The network in the medium-salinity group was the smallest in scale, with a high degree of centralization but a relatively simple structure. The high-salinity group network, of moderate scale, was characterized by higher density and complexity, resulting in a more compact network.

### 3.5. Trophic Types and Functional Groups of Fungi in Zhanjiang Bay Sediments

**Figure 6:**
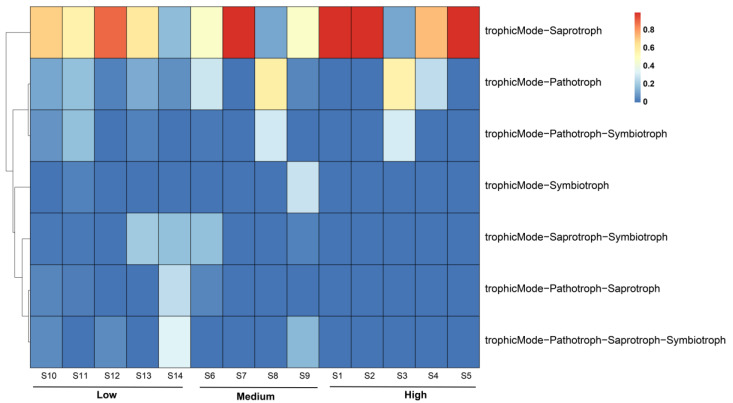
Functional prediction of nutritional modes of fungi in the sediments of Zhanjiang Bay, China (Low: low salinity; Medium: medium salinity; High: high salinity).

The nutritional types of the fungal communities in the sediments of Zhanjiang Bay were predicted using the FUNGuild database (Figure 6). The primary categories included saprotrophs, pathotrophs, symbiotrophs, pathotroph–symbiotrophs, saprotroph–symbiotrophs, pathotroph–saprotrophs, and pathotroph–saprotroph–symbiotrophs. Among these, saprotrophs exhibited the highest average relative abundance at 63.06%, followed by pathotrophs at 16.32%. In the high-salinity group, the average relative abundance of saprotrophs (76.65%) was significantly higher than that in the medium- (51.60%) and the low-salinity (58.64%) groups (*p* < 0.05). Conversely, the average relative abundance of pathotrophs was greater in the medium-salinity group (23.03%) compared to that in the low- (10.43%) and the high- (16.84%) salinity groups (Figure 6). For the pathotroph–saprotroph type, the relative abundance was higher in the low-salinity group than that in the medium- and the high-salinity groups. Other nutritional types, such as pathotroph–saprotroph–symbiotrophs, pathotroph–symbiotrophs, saprotroph–symbiotrophs, and symbiotrophs, were also present in different salinity groups, though their relative abundances were lower.

**Figure 7:**
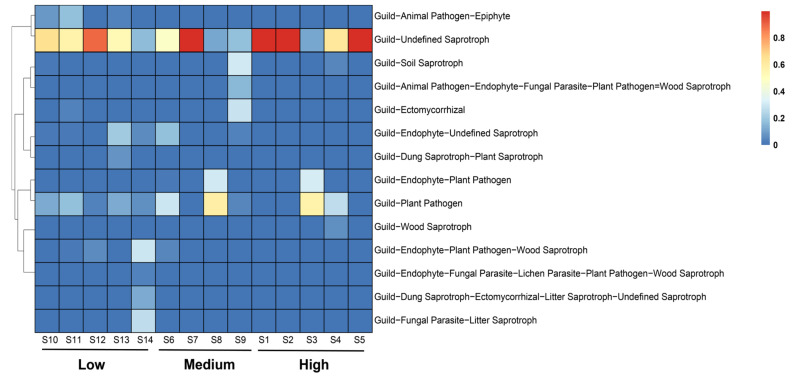
Ecological functional groups of fungi in the sediments of Zhanjiang Bay, China (Low: low salinity; Medium: medium salinity; High: high salinity).

The identified nutritional types were further classified into 14 ecological functional groups based on the fungi’s modes of resource absorption and utilization (Figure 7). The ecological functional groups with a relative abundance exceeding 1% and clearly defined functions were as follows: low-salinity group (Undefined Saprotroph, Plant Pathogen, Endophyte–Plant Pathogen, Endophyte–Undefined Saprotroph, and Dung Saprotroph–Plant Saprotroph), medium-salinity group (Undefined Saprotroph, Plant Pathogen, Endophyte–Plant Pathogen, Endophyte–Undefined Saprotroph, Soil Saprotroph, and Ectomycorrhizal), and high-salinity group (Undefined Saprotroph, Plant Pathogen, Endophyte–Plant Pathogen, and Wood Saprotroph). The three groups shared two common functional groups: Plant Pathogen and Undefined Saprotroph. Further analyses revealed significant differences in functional group distributions among the three salinity groups (*p* < 0.05; Figure 7). The functional group with the highest relative abundance was Undefined Saprotroph (66.39%). The high-salinity group exhibited a significantly higher average relative abundance of Undefined Saprotrophs (74.41%) compared to the medium- and the low-salinity groups (43.95% and 56.29%). Within the Plant Pathogen functional group, the medium-salinity group had a higher average relative abundance (23.03%) compared to the low- and the high-salinity groups (10.46% and 16.84%). Notably, the functional groups Animal Pathogen–Epiphyte and Fungal Parasite–Litter Saprotroph were found exclusively in the fungal communities of the low-salinity group. In contrast, the functional group Endophyte–Plant Pathogen was present only in the fungal communities of the medium- and high-salinity groups.

### 3.6. Correlation Analysis Between Fungi and Environmental Factors

**Figure 8:**
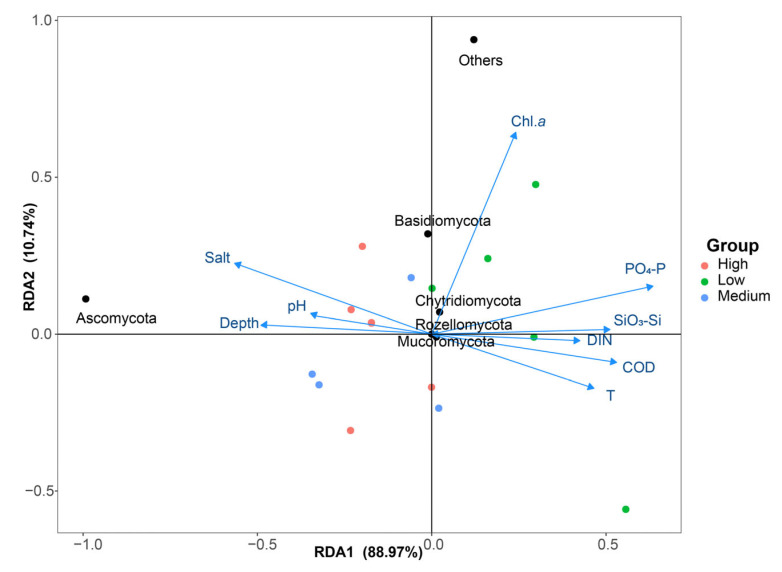
RDA of fungal community structure and environmental factors in the sediments of Zhanjiang Bay, China (Low: low salinity; Medium: medium salinity; High: high salinity).

The redundancy analysis (RDA) was performed to explore the relationship between the sampling sites and environmental factors using multivariate direct gradient regression analysis based on the environmental factors and ASVs (Figure 8). In the RDA plot, the environmental variables are represented by arrows, where the length of each arrow indicates the magnitude of its influence on community variation—longer arrows denote stronger effects. The angle between the arrows reflects the correlation between environmental variables: arrows pointing in the same direction indicate a positive correlation, while those pointing in opposite directions represent a negative correlation. The proximity of a sample point to an arrow represents the strength of the influence of that variable on the sample, with shorter distances indicating a stronger effect. The angle between the line connecting a sample point to the origin and the direction of an arrow signifies the correlation between the environmental variable and the sample data, where an acute angle indicates a positive correlation, and an obtuse angle indicates a negative correlation. RDA1 explained 88.97% of the variation in the sediment fungal community, while RDA2 accounted for 10.74% (Figure 8). The sampling points from the medium- and high-salinity groups were primarily located in the second and third quadrants, showing a positive correlation with salinity, pH, and depth. In contrast, the sampling points from the low-salinity group were more closely associated with variables such as DIN, PO_4_-P, and SiO_3_-Si. Among the fungal taxa, Ascomycota stood out as markedly different from the other groups, exhibiting a negative correlation with salinity, pH, and depth (Figure 8). The arrow representing salinity was the longest and formed the smallest angle with Ascomycota, signifying that salinity was the predominant factor influencing the Ascomycota fungal community. Basidiomycota showed a positive correlation with salinity, pH, and Chl *a*, and a negative correlation with the other environmental factors (Figure 8). Among these, the content of chlorophyll *a*, which reflects phytoplankton abundance, was the primary factor influencing the distribution of Basidiomycota fungal communities. Rozellomycota, Chytridiomycota, and Mucoromycota were positioned near the origin, indicating that these fungal communities were influenced by a combination of all the environmental factors.

## 4. Discussion

Traditional methods for studying microbial community structure, such as isolation and purification cultures, 16S rDNA denaturing gradient gel electrophoresis (DGGE), and 16S rDNA library approaches, have long been the mainstay in this field [40,41]. However, these conventional techniques are time-consuming, labor-intensive, and offer limited throughput. Given the vast diversity of marine sediment fungi and the complex, dynamic nature of their habitats, the data obtained through traditional methods are often insufficient and may lead to significant inaccuracies in the analyses. These limitations hinder the effective elucidation of the true structure of fungal communities. High-throughput sequencing technology, which bypasses the need for cultivation, isolation, and purification, directly sequences PCR-amplified products to generate extensive species information. This method is characterized by its high throughput and rapid sequencing capabilities, making it widely applicable in biodiversity research [42,43]. Previous studies on the biodiversity and distribution of fungal communities in the sediments of Zhanjiang Bay, China, remain limited. Accordingly, this study employed culture-independent ITS sequencing technology to investigate the composition and diversity of fungal communities in the sediments of Zhanjiang Bay, China. Additionally, the study sought to further elucidate the differences in fungal community characteristics across various salinity regions within the bay.

### 4.1. Fungal Community Composition and Diversity in Zhanjiang Bay Sediments

Zhanjiang Bay is a typical semi-enclosed bay, which hosts a microbial community structure that is highly complex and variable due to the influence of anthropogenic activities (e.g., marine engineering, aquaculture, etc.) and climate change [44]. Recent research has focused on microbial diversity in Zhanjiang Bay, while studies specifically addressing fungal diversity in the sediments remain scarce. In this study, ITS sequencing was employed to investigate the fungal communities in the sediments of Zhanjiang Bay, resulting in the identification of 1242 ASV sequences, representing 144 genera and five phyla. Ascomycota and Basidiomycota were identified as the dominant phyla of fungal communities in the sediments of Zhanjiang Bay, China. Among these, Ascomycota was overwhelmingly predominant, accounting for 68.97% of the relative abundance, followed by Basidiomycota with 6.41%. These findings are consistent with previous studies, for example, Wu et al. found that Ascomycota (80.02%) was the predominant phylum of fungi, followed by Basidiomycota (14.86%) in the coastal sediments from Guangdong, China [17]. Taylor et al. investigated fungal community structure in the western English Channel and identified Ascomycota and Basidiomycota as the two most dominant phyla [45]. Similarly, Wang et al. found that Ascomycota and Basidiomycota were the predominant marine fungi in semi-enclosed shallow bay ecosystems along the coast of the Bohai Sea, China [46]. Lin et al. also reported that Ascomycota and Basidiomycota were the most predominant groups of fungal diversity in typical coastal sediments of China seas [47]. From the perspective of species composition at different sampling sites, the relative abundances of Ascomycota and Basidiomycota in the moderate- to high-salinity group were higher than those in the low-salinity group. This pattern may be attributed to the halotolerant and halophilic characteristics of many species within the two phyla [48]. Additionally, both phyla exhibited a high degree of environmental adaptability and were often positively correlated with the concentration of organic pollutants in the environment, functioning as decomposers that played a significant role in the carbon mineralization process within marine ecosystems [49,50]. Our findings and previous research on marine fungal diversity collectively highlighted the widespread distribution of Ascomycota and Basidiomycota in the marine ecosystems. Moreover, 1.61% of the sequences were identified as belonging to the phyla Chytridiomycota, Rozellomycota, and Mucoromycota. However, these fungi were not detected in all the samples, suggesting that these low-frequency fungi were not widely distributed in the sediment environment of Zhanjiang Bay and may fulfill specific ecological roles. Additionally, 23.01% of the fungal ASV sequences could not be assigned to any known fungal phyla based on the existing database, which may be attributed to the insufficient coverage of ITS sequences in the current databases. A similarly high proportion of unidentified fungi has been reported in other marine sediments, indicating the existence of a substantial number of unknown fungal groups in marine sediment environments [51,52,53].

Biodiversity indices are essential for assessing microbial communities, as microbial diversity is crucial for maintaining the functionality of these communities in dynamic environments [54]. Significant differences in the diversity of the fungal communities in the sediments across the low-, medium-, and high-salinity regions of Zhanjiang Bay have been observed. These variations may be attributed to differences in nutrient levels and fungal dispersal capabilities, which are likely influenced by human activities and the distinct geographical characteristics of these regions [55]. Resource availability also plays a critical role in shaping fungal diversity, and fungal communities are particularly sensitive to deterministic processes [56]. Both spatial and environmental factors significantly influence the distribution of marine fungi [57]. The Shannon index and Richness index of the fungal communities in the sediments of Zhanjiang Bay are significantly higher in the low-salinity group compared to the other two groups. This heightened diversity may be linked to the presence of numerous residential areas in the low-salinity region, where human activities such as agricultural fertilization, livestock farming, industrial wastewater discharge, and domestic sewage introduce substantial amounts of nitrogen (N) and phosphorus (P) into the marine environment. The resulting increase in nutrient concentrations, particularly nitrogen and phosphorus, likely influences the structure and diversity of marine microbial communities. This effect may also be related to the topographical features of the Zhanjiang Bay. The bay mouth, serving as the entry point to the sea, exhibits relatively higher salinity, while the northern part of the bay, which receives inflows from rivers such as the Suixi River, has relatively lower salinity. Salinity is a critical environmental factor influencing the survival, growth, and distribution of microorganisms in marine ecosystems. Wang et al. demonstrated that salinity is a key factor affecting the diversity of coastal fungal communities [58]. Similarly, Abdel-Gawad et al. found that seawater temperature, salinity, and pH are the most significant environmental factors influencing fungal diversity in the Red Sea of Egypt, showing that temperature and salinity may impact the growth rate and metabolic activity of fungal communities [59]. Zhang et al. further demonstrated that fungal diversity is primarily influenced by salinity, organic carbon, silicate, and phosphate concentrations in Arctic sediments [60].

### 4.2. Fungal Community Molecular Network Analysis in Zhanjiang Bay Sediments Based on ITS Metabarcoding

The molecular ecological network provides a powerful framework for assessing the complexity and stability of microbial communities [61]. Parameters such as connectivity, degree, and modularity within the network’s topological structure are more sensitive indicators of environmental changes than traditional microbial diversity indices [62]. In molecular ecological networks, increased connectivity, degree, average clustering coefficient, relative modularity, and the number of key species are indicative of a more complex and stable network [63]. In recent years, the application of molecular ecological network analysis has become increasingly prevalent in microbial ecology research [64,65]. Accordingly, we employed the construction of molecular network topologies to analyze the complexity and stability of the fungal communities in the sediments of Zhanjiang Bay. The results revealed that the molecular ecological network in the low-salinity group had a greater number of nodes compared to the medium- and high-salinity groups, while the network density and average clustering coefficient were lower in the low-salinity group. This suggests that the network in the low-salinity group is larger and more dispersed, whereas the fungal communities in the sediments of the medium- and high-salinity groups in Zhanjiang Bay exhibit closer connections. In summary, salinity has a significant impact on the structure of the molecular ecological network of the fungal communities in the sediments of Zhanjiang Bay. When salinity increases, the compactness and complexity of the network also increase. Additionally, the molecular modularity of the fungal communities in the low-salinity-group sediments (0.845) is higher than that in the medium-salinity (0.428) and high-salinity (0.834) groups. Studies have shown that a higher degree of network modularity can mitigate the impact of environmental changes on the molecular ecological network, thereby enhancing the network’s stability to some extent [63]. Compared to the low-salinity group, the networks in the medium- and high-salinity groups are relatively smaller, indicating that under extreme conditions, fungal diversity and interaction frequency significantly decrease. This reduction may be attributed to increased salinity, which limits the survival and reproduction of fungi, thereby affecting their ecological interactions. Wang et al. found that low salinity and high nutrient conditions favor fungal growth, whereas high salinity and low nutrient levels tend to restrict it [58]. Similarly, Burgaud et al. observed that fungal abundance was higher in lower-salinity environments [19]. In this study, the fungal groups primarily involved in the network connections were Ascomycota, Basidiomycota, Chytridiomycota, and Rozellomycota, while the other fungal phyla and unannotated species had a relatively low representation in the network construction process. These fungi are widely distributed in marine environments, and their diversity is closely linked to ecosystem stability. The diversity of key fungal groups can enhance the overall stability of the ecosystem [66]. Therefore, analyzing these network relationships provides valuable insights into the role of fungi within the ecosystem, offering important guidance for ecological conservation and sustainable development.

### 4.3. Potential Functional Traits of Fungal Communities in the Sediments of Zhanjiang Bay

FUNGuild is a comprehensive database that facilitates the comparison of fungal trophic types and ecological functions, enabling the identification of fungal trophic strategies and the prediction of their specific roles [67]. However, several limitations arise when applying this tool to the analysis of marine fungal diversity. First, FUNGuild’s database is predominantly based on fungal data from terrestrial ecosystems, resulting in a limited representation of marine fungal species and their specific functions, which constrains its applicability in marine environments [68]. Second, while FUNGuild classifies fungi into functional groups such as decomposers, symbionts, and pathogens, marine fungi may exhibit distinct functional roles or possess characteristics that do not fully align with these predefined categories [69]. Additionally, FUNGuild infers functional roles based on the established functional information; however, the functions of marine fungi may vary in response to environmental changes, a dynamic that FUNGuild is unable to capture [70]. Overall, FUNGuild has high application value in terrestrial ecosystems. As highlighted in several studies, many marine fungi share phylogenetic lineages with terrestrial fungi, and functional similarities have been observed [71,72]. Although research on marine fungi is still in its nascent stages, the existing databases and algorithms, such as FUNGuild, UNITE, and SILVA, are increasingly being employed for preliminary annotations and to explore marine fungal diversity and potential ecological functions [73]. Fungal trophic types are primarily classified into pathotrophs, symbiotrophs, and saprotrophs; pathotrophic fungi acquire nutrients by damaging host cells, symbiotrophic fungi obtain nutrients through resource exchange with host cells, and saprotrophic fungi obtain nutrients by decomposing dead plant or animal matter [41]. FUNGuild predictions indicate that the fungi in the three study areas are predominantly saprotrophic and pathotrophic, and this is likely due to the dominance of Ascomycota and Basidiomycota there. Many fungi within the Ascomycota phylum are saprotrophic, efficiently decomposing and degrading organic matter [74]. Meanwhile, fungi in the Basidiomycota phylum are primarily saprotrophic or parasitic, with the ability to break down recalcitrant substances such as lignocellulose [75]. These fungal functional groups play a crucial role in converting macromolecular organic matter into smaller molecules in the soil. Some of these smaller molecules are utilized by the fungi themselves and other organisms, while the remaining organic substances are released into the sediment, thereby increasing the organic matter content in the sediment. In this study, the relative abundance of saprotrophic fungi in the high-salinity-group sediments was 76.65%, significantly higher than that in the medium-salinity group (58.64%) and the low-salinity group (51.60%). The average relative abundance of pathotrophs in the medium-salinity group (23.03%) exceeded that in both the low-salinity group (10.43%) and the high-salinity group (16.84%). Additionally, the average relative abundance of pathotroph–saprotroph fungi was highest in the low-salinity group (8.17%) compared to the medium-salinity group (5.39%) and the high-salinity group (0.07%). Furthermore, pathotroph–saprotroph–symbiotroph, pathotroph–saprotroph, and saprotroph–symbiotroph types were found exclusively in the fungal communities of the high-salinity-group sediments. These findings suggest that fungi may adopt different trophic strategies to thrive in varying habitats.

### 4.4. Environmental Drivers of Fungal Community Structure in the Sediments of Zhanjiang Bay

With ongoing rapid economic development, terrestrial pollutants such as nitrogen, phosphorus, and organic contaminants increasingly enter the ocean through land runoff, leading to the accumulation of substantial amounts of pollutants [76,77]. This process alters the physicochemical properties of seawater, inflicting damage on marine ecosystems and subsequently affecting the diversity of marine microorganisms [77]. Marine fungi, which play a critical role in material metabolism and energy flow processes, are closely linked to environmental factors and can rapidly respond to changes within the marine environment [66]. The key environmental factors influencing these dynamics include water temperature, salinity, nitrogen, phosphorus, and COD [78]. In this study, the salinity in Zhanjiang Bay exhibited a decreasing trend from south to north and from the bay mouth to the inner bay, which is consistent with previous studies. For instance, Jiang et al. used observational data from October 2015 to conclude that salinity in the Zhanjiang Port sea area gradually increases horizontally from the inner bay to the outer bay [79]. Similarly, Huang et al. also found that salinity progressively increases from the bay head, through the bay neck, dike, and shoal areas, to the bay mouth in Zhanjiang Bay [80]. This pattern is primarily influenced by the topographic characteristics of Zhanjiang Bay, specifically, the bay mouth opening to the sea, which exhibits relatively high salinity, whereas the northern part of the bay, influenced by the confluence of Suixi River, experiences relatively lower salinity.

A redundancy analysis (RDA) was conducted on the fungal communities in Zhanjiang Bay considering eight environmental factors, e.g., salinity, temperature, COD, etc. As the sampling reason, the environmental factors in the bottom waters could not be obtained by our instruments. Therefore, the analyses of the correlation between the fungal community structure and environmental factors were based on surface water environmental factors, as there was no stratification in Zhanjiang Bay in April, and very little difference in the environmental factors in the surface and bottom waters. In addition, the physicochemical properties of the surface water (such as temperature, pH, dissolved oxygen, salinity, and nutrients) had a direct impact on the microbial communities in the sediment. The results indicated that salinity is the primary factor influencing the Ascomycota fungal community, consistent with previous research findings. Gunde-Cimerman et al. observed that many species within the Ascomycota phylum exhibited halotolerant and halophilic characteristics [48]. In marine environments, a key ecological function of Ascomycota is the decomposition of organic matter, including plant debris, animal remains, and other organic materials [66]. This function is particularly crucial in high-salinity environments, where other microorganisms may be constrained by the elevated salt conditions. In such environments, Ascomycota typically synthesizes and accumulates organic osmolytes (such as glycerol, betaine, etc.) to balance osmotic pressure, thereby preventing cellular dehydration and damage [81]. Conversely, in lower-salinity environments such as estuaries, the relative abundance of Ascomycota may be reduced, allowing other low-salinity-tolerant or non-halophilic fungal groups to become more dominant [82]. Basidiomycota, Chytridiomycota, Mucoromycota, and Rozellomycota are significantly influenced by environmental factors such as chlorophyll *a*, COD, pH, and temperature, which is consistent with previous studies. Ahumada-Rudolph et al. found that changes in environmental pH can impose physiological constraints on fungal growth and reproduction, thereby affecting the diversity of fungal communities [83]. Similarly, Huang et al. identified COD as a key driving factor influencing the fungal community structure in the Dafengjiang Estuary [84]. Sun et al. found that fungi can play a pivotal role in the occurrence and development of various phytoplankton blooms in aquatic ecosystems [85]. Additionally, Basidiomycota, Chytridiomycota, Mucoromycota, and Rozellomycota are also affected by environmental factors such as phosphate, silicate, and DIN. Nitrogen and phosphorus are the key limiting factors for the growth of marine fungi, influencing the metabolic activities and physiological characteristics of fungal communities. Wang et al. found that an increase in the environmental nitrogen content exerts an inhibitory effect on the growth of *Aspergillus flavus* [86]. Similarly, Taylor et al. identified nitrogen availability as the primary environmental factor driving the structure of fungal communities along the western coast of the English Channel [45]. Additionally, Wang et al. reported that dissolved inorganic phosphorus, silicate, and inorganic nitrogen strongly influenced the fungal community structure in the Bohai Sea, China [58]. These findings suggest that fluctuations in nutrients such as nitrogen and phosphorus in the marine environment can significantly alter the community structure and species composition of marine fungi. Therefore, studying the relationship between nutrients and marine fungi is crucial for understanding nutrient dynamics within marine ecosystems and the critical role that fungi play in these processes. This understanding is essential for the effective protection and management of marine resources, as well as for responding to environmental changes.

## 5. Conclusions

This study conducted a comprehensive analysis of the fungal community structure within the sediments of Zhanjiang Bay, China, revealing that the community is predominantly composed of the phyla Ascomycota, Basidiomycota, Rozellomycota, Chytridiomycota, and Mucoromycota, with Ascomycota and Basidiomycota emerging as the dominant groups. Significant variations in species composition and diversity, as measured by α-diversity and β-diversity indices, were observed across the three study areas. Notably, the α-diversity index for fungi in the low-salinity group was significantly higher than that in the other two groups. The functional prediction analysis using FUNGuild categorized the majority of fungal species into the nutritional types of symbiotroph, saprotroph, and pathotroph, and further subdivided them into 14 ecological functional groups. The fungal community structure was found to be primarily influenced by surface water environmental factors such as temperature, salinity, COD, and DIN, with salinity exhibiting the strongest correlation, thus identified as the most critical environmental driving factor. This research fills a significant gap in the current understanding of fungal community structures within the sediments of Zhanjiang Bay, China, offering valuable insights into the region’s biodiversity of protists. Additionally, it provides essential scientific data and a robust evidence base to support ongoing and future environmental protection initiatives in Zhanjiang Bay, China.

## Figures and Tables

**Table 1:** Physicochemical variables at the sampling stations in Zhanjiang Bay, China.

Station	Depth (m)	T (°C)	pH	Salt	Chl-*a* (µg/L)	DIN (mg/L)	SiO_3_-Si (mg/L)	PO_4_-P (mg/L)	COD (mg/L)
S1	15.4	24.63	7.65	30.63	0.49	0.240	1.408	0.008	0.88
S2	27.3	24.87	7.72	31.67	0.49	0.231	1.387	0.003	1.36
S3	36.4	24.74	7.71	32.07	0.49	0.234	1.538	0.002	1.12
S4	9.6	25.13	7.69	32.01	1.47	0.238	1.565	0.030	1.04
S5	7.9	24.84	7.70	31.32	1.47	0.209	1.470	0.004	0.96
S6	4.9	24.64	7.68	28.75	0.98	0.281	1.626	0.045	1.28
S7	6.8	24.65	7.67	30.01	1.47	0.262	1.385	0.014	0.96
S8	10.6	24.08	7.67	30.12	1.47	0.278	1.385	0.042	1.20
S9	6.6	24.66	7.65	29.92	1.97	0.274	1.646	0.027	1.20
S10	18.5	24.15	7.63	29.18	1.97	0.348	2.123	0.066	1.20
S11	14.3	24.29	7.61	29.08	3.44	0.302	1.894	0.089	1.28
S12	7.7	24.74	7.70	27.56	1.47	0.381	2.790	0.094	1.44
S13	7.2	25.07	7.64	24.44	0.49	0.340	2.451	0.071	1.60
S14	2.1	24.64	7.50	18.48	2.95	0.770	5.428	0.117	2.96

T: temperature; pH: pH value; Salt: salinity.

## Data Availability

The original contributions presented in the study are included in the article/Supplementary Materials, further inquiries can be directed to the corresponding author.

## Supplementary Materials

The following supporting information can be downloaded at https://www.mdpi.com/article/10.3390/jof10120867/s1. Figure S1. Chao1 rarefaction curves of the 14 samples. Figure S2. Observed ASVs rarefaction curves of the 14 samples. Figure S3. Shannon rarefaction curves of the 14 samples. Figure S4. Simpson rarefaction curves of the 14 samples. Figure S5. Goods coverage rarefaction curves of the 14 samples. Figure S6. Species composition and relative abundance of fungal communities in sediments of Zhanjiang Bay, China (Class level). Figure S7. Molecular ecological networks of fungal communities in high salinity group sediments of Zhanjiang Bay, China. Figure S8. Molecular ecological networks of fungal communities in low salinity group sediments of Zhanjiang Bay, China. Figure S9. Molecular ecological networks of fungal communities in medium salinity group sediments of Zhanjiang Bay, China. Table S1. Statistics of obtained sequence data of fungal ITS gene amplicon pyrosequencing. Table S2. Alpha diversity estimates of fungal communities in 14 samples. Table S3. Annotation statistics of different classification levels for 14 samples. Table S4. Annotations of 1242 ASVs obtained in this study. Table S5. Topological parameters of the molecular ecological network of fungal communities in the sediments of Zhanjiang Bay, China.
