# Supplementary material for: Diversity and Distribution of Fungi in the Marine Sediments of Zhanjiang Bay, China
Source: J Fungi (Basel). 2024 Dec 13;10(12):867. doi: 10.3390/jof10120867 (PMC11677748; doi:10.3390/jof10120867)
Supplement: Supplementary file 1 [file jof-10-00867-s001.zip › Supplementary Figures.pdf]

Supplementary Figures

Supplementary Figure S1. Chao1 rarefaction curves of the 14 samples.

Supplementary Figure S2. Observed ASVs rarefaction curves of the 14 samples.

Supplementary Figure S3. Shannon rarefaction curves of the 14 samples.

Supplementary Figure S4. Simpson rarefaction curves of the 14 samples.

Supplementary Figure S5. Goods coverage rarefaction curves of the 14 samples.

Supplementary Figure S6. Species composition and relative abundance of fungal communities in sediments of Zhanjiang Bay, China (Class level).

Supplementary Figure S7. Molecular ecological networks of fungal communities in high salinity group sediments of Zhanjiang Bay, China.

Supplementary Figure S8. Molecular ecological networks of fungal communities in low salinity group sediments of Zhanjiang Bay, China

Supplementary Figure S9. Molecular ecological networks of fungal communities in medium salinity group sediments of Zhanjiang Bay, China

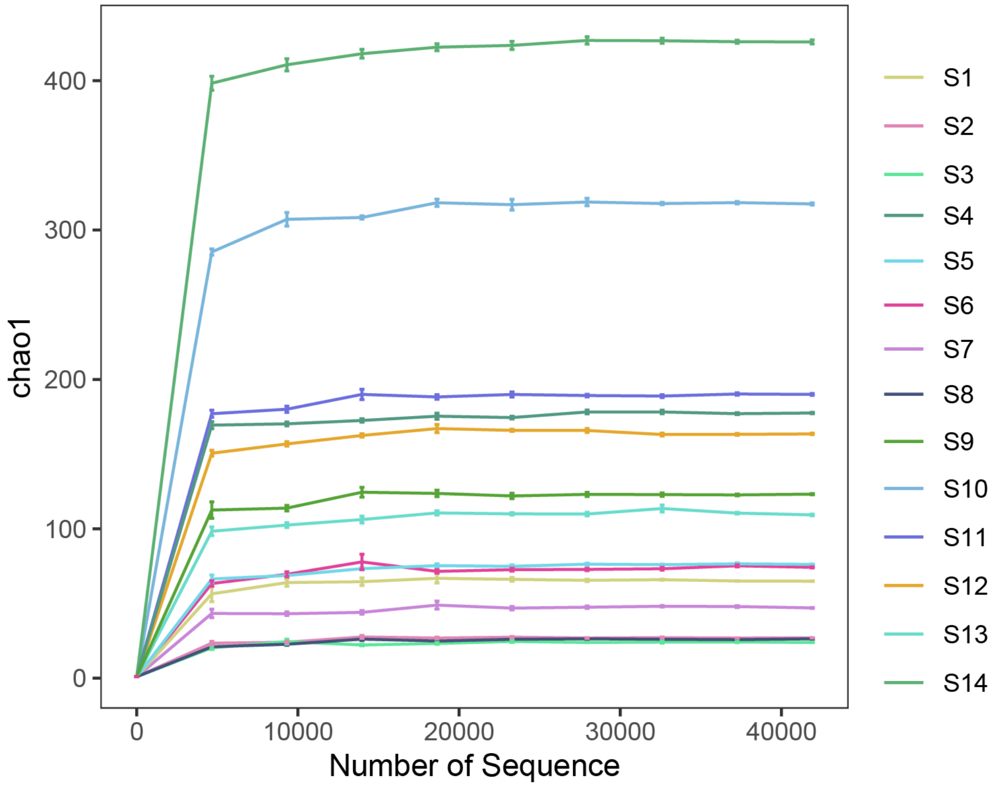

Supplementary Figure S1. Chao1 rarefaction curves of the 14 samples.

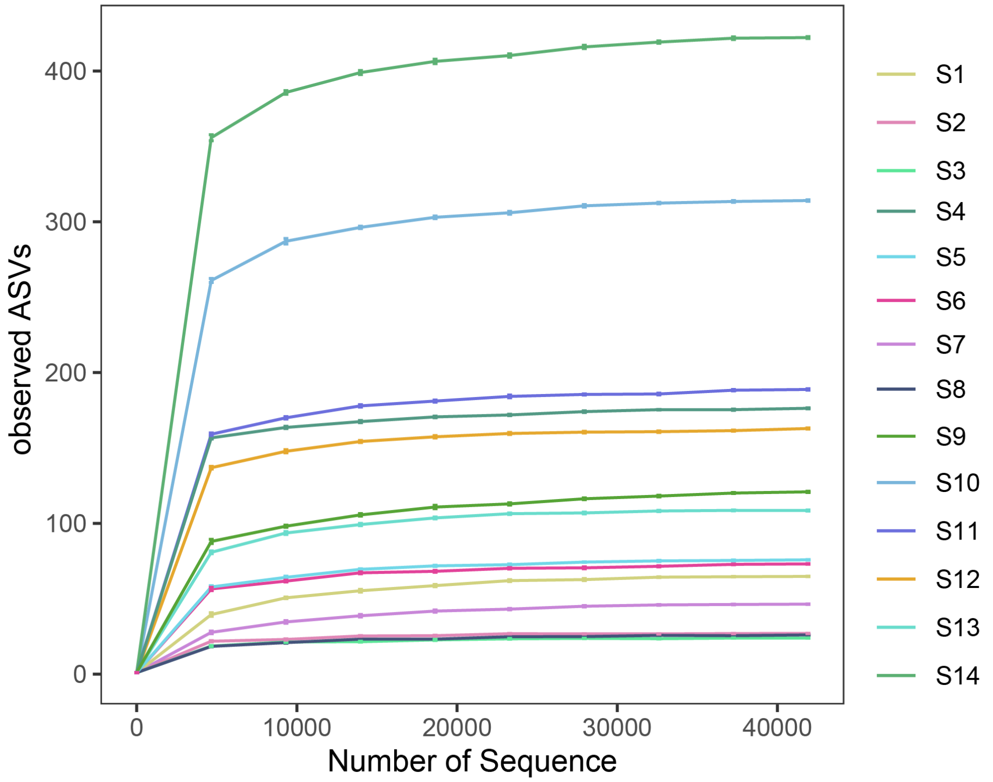

Supplementary Figure S2. Observed ASVs rarefaction curves of the 14 samples.

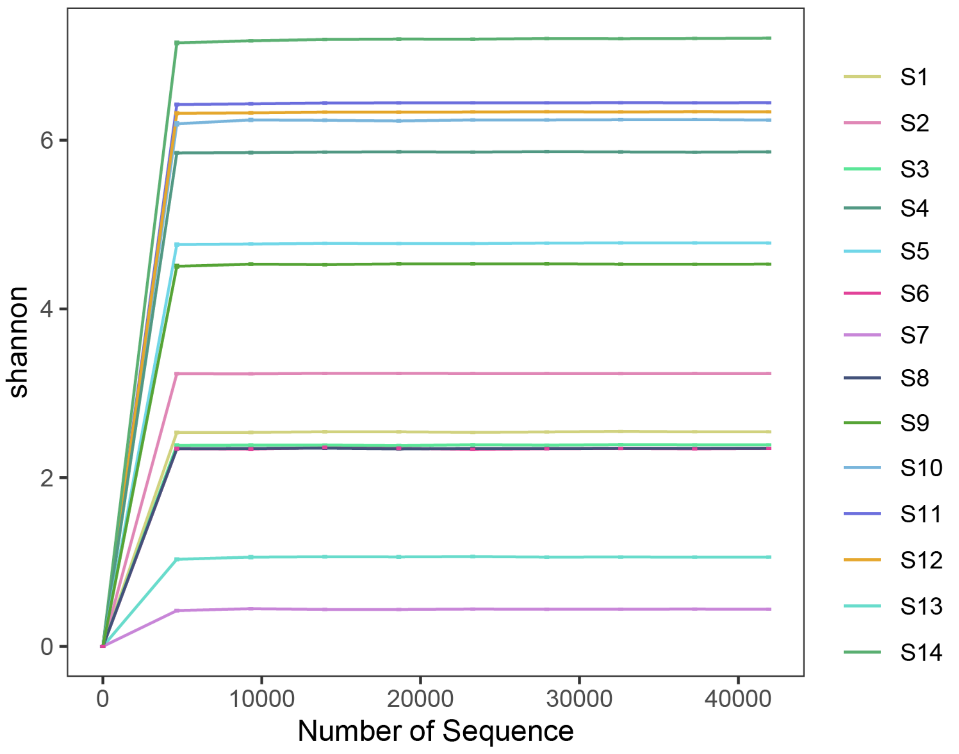

Supplementary Figure S3. Shannon rarefaction curves of the 14 samples.

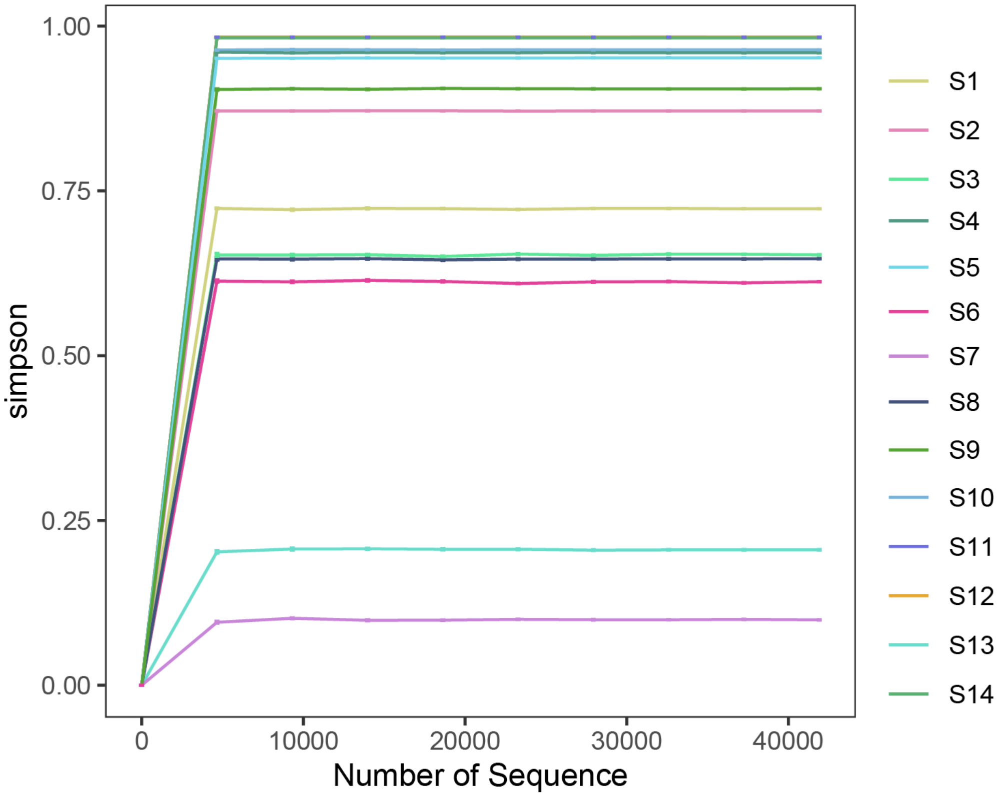

Supplementary Figure S4. Simpson rarefaction curves of the 14 samples.

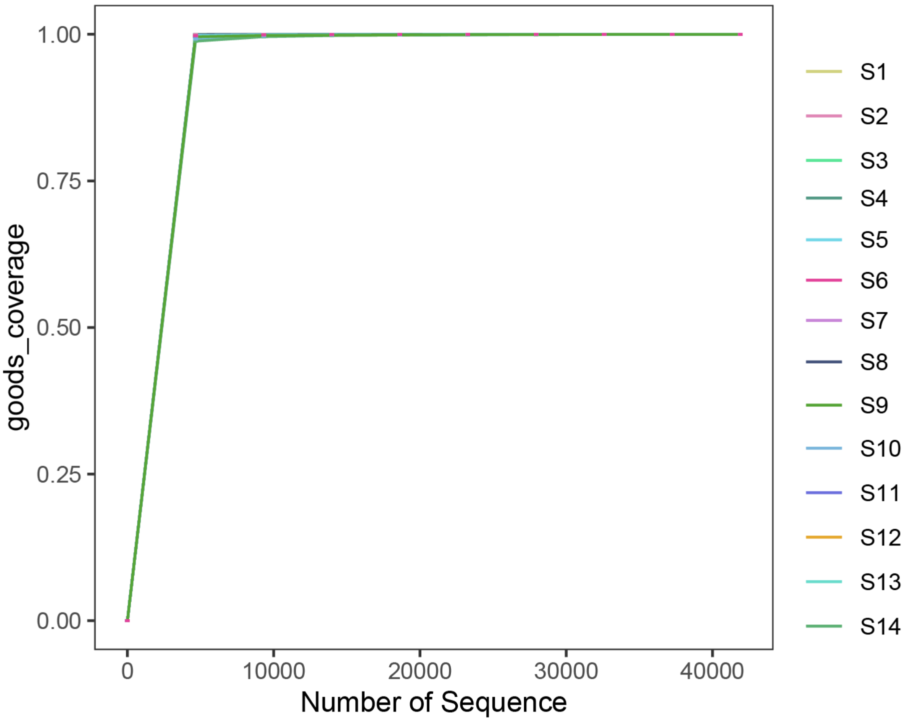

Supplementary Figure S5. Goods coverage rarefaction curves of the 14 samples.

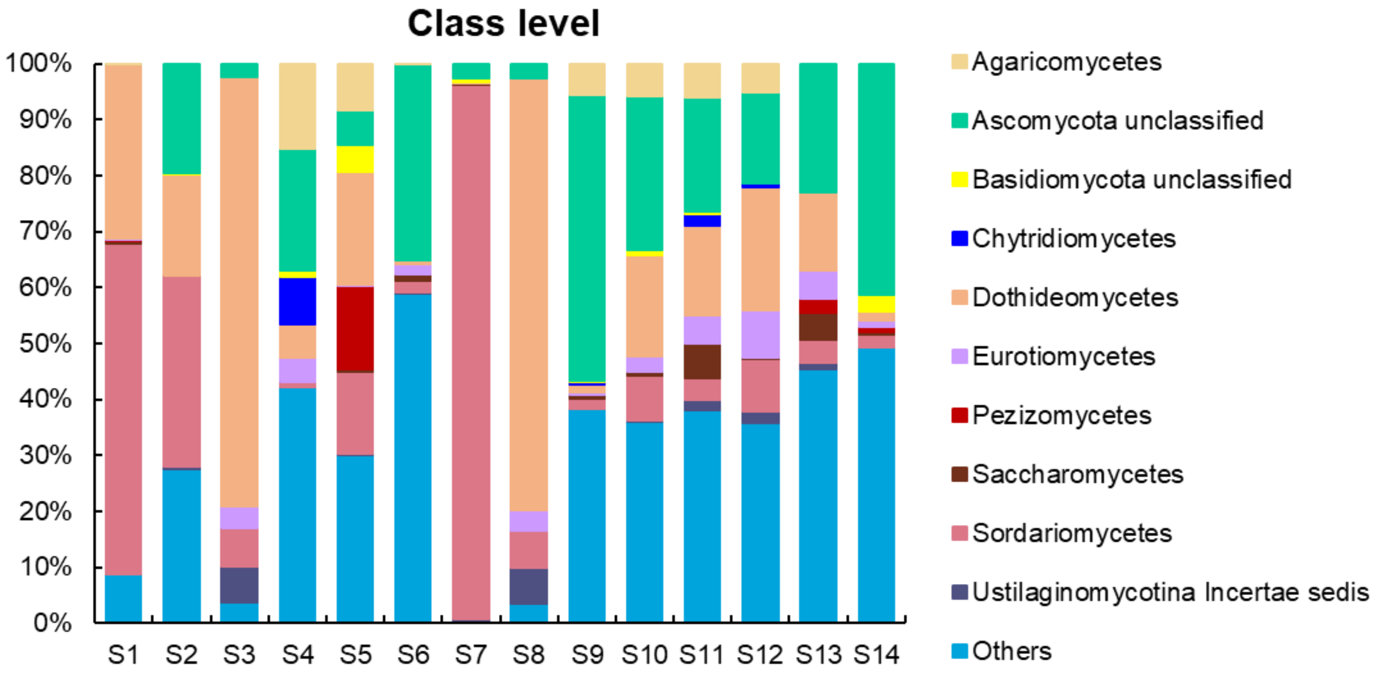

Supplementary Figure S6. Species composition and relative abundance of fungal communities in sediments of Zhanjiang Bay, China (Class level).

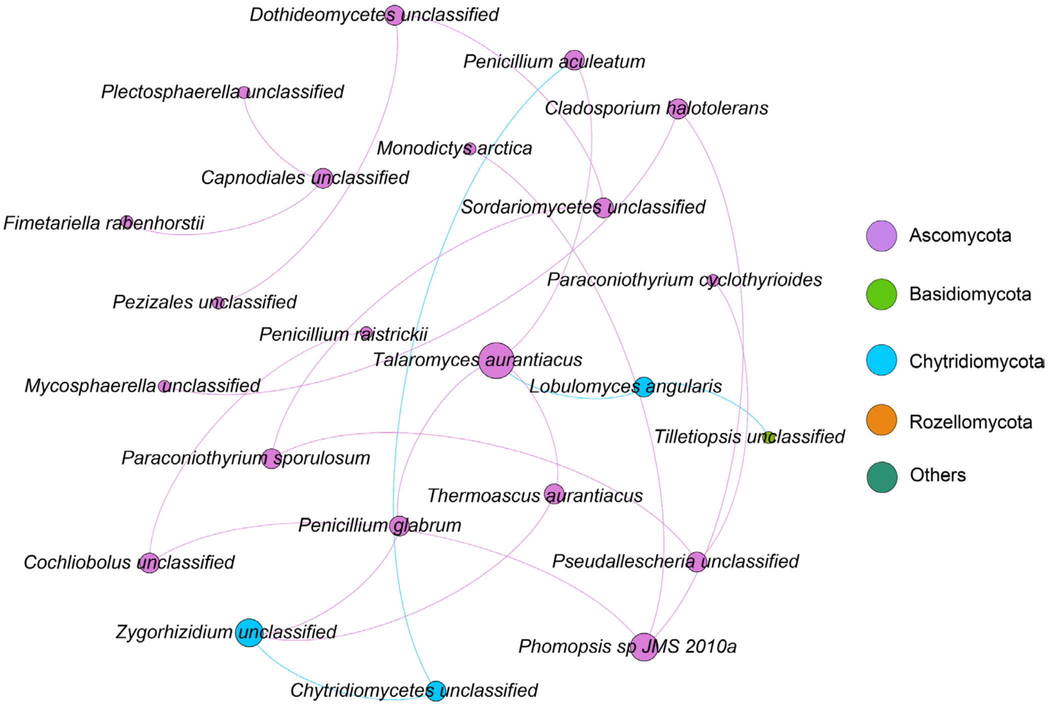

Supplementary Figure S7. Molecular ecological networks of fungal communities in high salinitygroup sediments of Zhanjiang Bay, China.

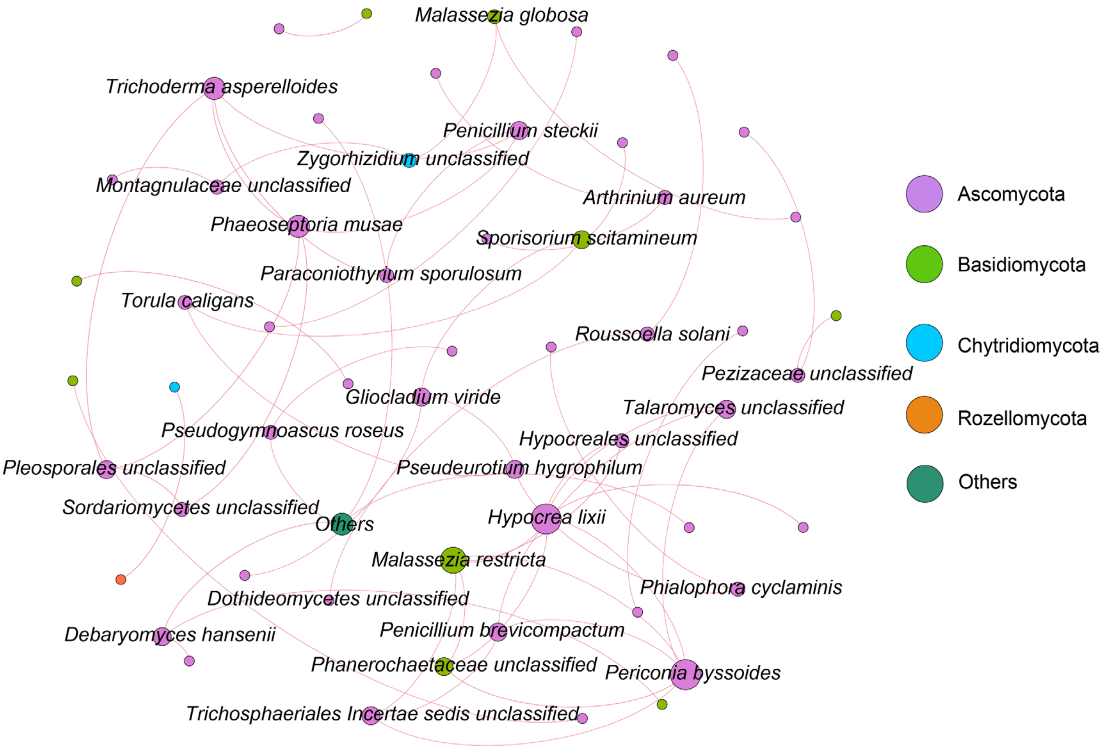

Supplementary Figure S8. Molecular ecological networks of fungal communities in low salinity group sediments of Zhanjiang Bay, China

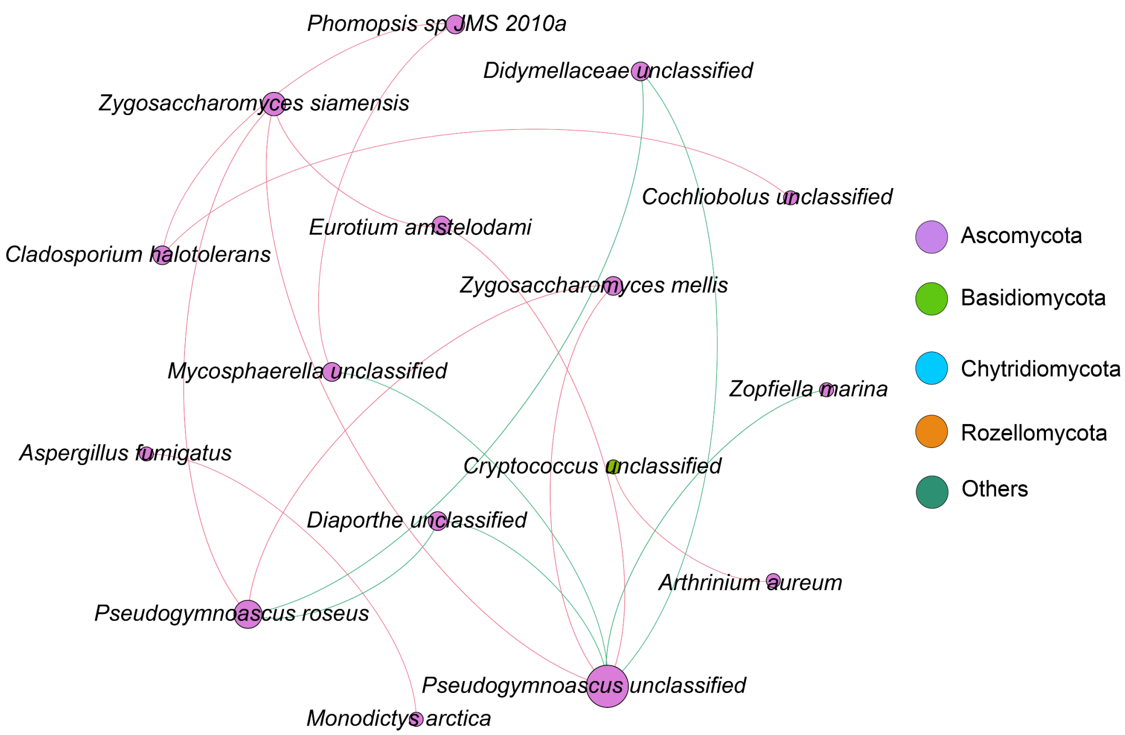

Supplementary Figure S9. Molecular ecological networks of fungal communities in medium salinity group sediments of Zhanjiang Bay, China
